# Structural and Magnetic Properties of Perovskite Functional Nanomaterials La_1*−x*_R*_x_*FeO_3_ (R = Co, Al, Nd, Sm) Obtained Using Sol-Gel

**DOI:** 10.3390/molecules28155745

**Published:** 2023-07-29

**Authors:** Fang Yang, Xingxing Yang, Kaimin Su, Jinpei Lin, Yun He, Qing Lin

**Affiliations:** 1 College of Biomedical Information and Engineering, Hainan Medical University, Haikou 571199, China; 2 College of Physics and Technology, Guangxi Normal University, Guilin 541004, China; 3 Department of Civil Engineering, Jiangxi Water Resources Institute, Nanchang 330013, China

**Keywords:** functional nanomaterials, structure, magnetic, perovskite, substituted, Sol-gel

## Abstract

Perovskite is the largest mineral on earth and has a variety of excellent physical and chemical properties. La_1*−x*_R*_x_*FeO_3_ (R = Co, Al, Nd, Sm) were synthesized using the sol-gel method and analyzed by XRD, TG-DTA, and VSM. With the increase in the Co^2+^ doping content, the diffraction peak drifted in the direction of a larger angle. The grain size of La_1*−x*_R*_x_*FeO_3_(R = Co) is mainly concentrated between 50.7 and 133.5 nm. As the concentration of Co^2+^ increased, the magnetic loop area and magnetization increased. La_1*−x*_R*_x_*FeO_3_(R = Al) is an orthorhombic perovskite structure, the grain size decreased with the increase in Al^3+^ doping concentration, and the minimum crystallite is 17.9 nm. The magnetic loop area and magnetization increased with the increase in Al^3+^ ion concentration. The enclosed area of the M-H curve of the sample decreased, and the ferromagnetic order gradually weakened and tended to be antiferromagnetic, which may be due to the increase in sintering temperature, decrease in the iron oxide composition, and changes in the magnetic properties. Proper doping can improve the magnetization of La_1*−x*_R*_x_*FeO_3_(R = Nd), refine the particles, and obtain better magnetic performance. As the Nd^3+^ ion concentration increased, the magnetic properties of the samples increased. Ms of La_0.85_Co_0.15_FeO_3_ prepared by different calcination time increases with the increase in calcination time. As the Sm^3+^ ion concentration increased, the magnetic properties of the samples increased. Proper doping can improve the magnetization of La_1*−x*_R*_x_*FeO_3_(R = Sm), refine the particles, and generate better magnetic performance.

## 1. Introduction

Perovskite is the largest mineral on earth and has a variety of excellent physical and chemical properties, such as magnetoelectric effect, magnetostriction, variable magnetic phase transition, catalytic activity, and piezoelectric effect. It is one of the new functional materials with great development prospects [1,2]. LaFeO_3_ oxides with perovskite structure have a unique crystal structure; a slight change in its structure, especially defects in the crystal structure or changes in the performance caused by doping, will lead to new properties [3,4]. In the perovskite structure oxide ABO_3_, the A-site is generally a rare earth element with large radius (i.e., La, Pr, and Gd). The interaction between A-site ions and oxygen ions plays a decisive role in the mutual evolution of perovskite structures [5,6]. Lili Liu et al. [7] studied synthesis and characterization of Al^3+^ doped LaFeO_3_ compounds. Sr-doped porous LaFeO_3_ samples were fabricated via the sol-gel method [8]. Nabasmita Saikia et al. [9] prepared (La_0.75_Gd_0.25_)FeO_3_ successfully via the typical solid casting route method. Chethana Aranthady et al. [10] studied the Ca substituted LaFeO_3_. Yongfang Rao et al. [11] prepared Cu-doped LaFeO_3_ samples and studied heterogeneous catalysts of PMS for the degradation of pharmaceuticals. Wankassama Haron et al. [12] studied the structural characteristics and dielectric properties of La_1*−x*_Co*_x_*FeO_3_ and LaFe_1*−x*_Co*_x_*O_3_ samples using the thermal decomposition method. Xiutao Ge et al. [3] studied the gas sensitivity of LaFe_1−_*_y_*Co*_y_*O_3_ via the co-precipitation method. Yutana Janbutrach et al. [5] synthesized La_1*−x*_Al*_x_*FeO_3_ nanocrystals and studied their magnetic and optical properties. The saturation magnetization, the coercivity, and the remanent magnetization of the samples increase with the increase in the concentration of Al^3+^ ion. There are few studies that investigate the Al^3+^-doped LaFeO_3_ nanoparticles synthesized at low temperature using the citric acid sol-gel method and their structure and magnetic properties. LI Fa-tang et al. [1] synthesized La_1*−x*_Nd*_x_*FeO_3_ via the citric acid complexation method and studied its morphology. Enrico traversa et al. [2] synthesized La_1*−x*_Sm*_x_*FeO_3_ sample via the thermal decomposition method and studied its structure and composition. In this paper, La_1*−x*_R*_x_*FeO_3_ (R = Co, Al, Nd, Sm) powder was synthesized using the citric acid sol-gel method [13,14,15,16,17,18,19,20], and the influences of different Co, Al, Nd, and Sm doping ratios and calcination temperatures on the structure, morphology, and magnetic properties of the samples were studied.

## 2. Results and Discussion

### 2.1. XRD Analysis of La_1−x_R_x_FeO_3_ (R = Co)

**Figure 1:**
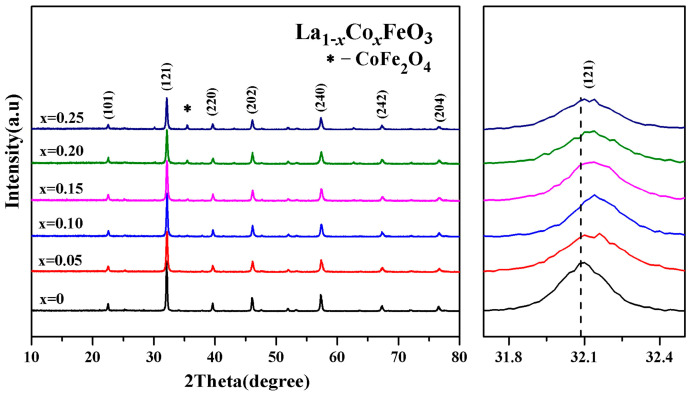
XRD diffraction and (121) peak drift patterns of La_1*−x*_Co*_x_*FeO_3_ (*x* = 0~0.25) samples calcined at 700 °C for 6 h.

Figure 1 is the XRD diffraction pattern of La_1*−x*_Co*_x_*FeO_3_ (*x* = 0~0.25). The XRD diagram can analyze the structural changes caused by atomic doping. When the doping amount *x* = 0.20, the samples showed a CoFe_2_O_4_ mixed phase, indicating that with the increase in Co^2+^ doped, Co^2+^ does not fully enter the La^3+^ node, and part of Co^2+^ and Fe^3+^ form a CoFe_2_O_4_ compound, which will decrease the magnetic properties of the samples.

The diffraction peak (121) intensity is decreased with the increase in Co^2+^ doping concentration. The diffraction peak (121) is wider. The crystallinity of the sample is reduced [21,22,23,24]. The drift pattern shows that with the increase in the Co^2+^ doping amount, the diffraction peak (121) drifted in the direction of a larger angle. Wankassama Haron [12] came to a similar conclusion. Wankassama Haron et al. [12] explained that the substitution of small radius Co^2+^ (ionic radius of 0.074 nm) for larger radius La^3+^ (ionic radius 0.274 nm) will reduce the c/a. Therefore, the lattice spacing d decreased. According to the Bragg diffraction condition 2dsinθ = nλ, it is known that diffraction peak drifted in the direction of 2θ increased. This explains that according to the equation below, the smaller radius of Co^3+^ (ionic radius of 0.167 nm) replaced the larger radius of La^3+^ (ionic radius of 0.274 nm) [14,15,16]:

(1) d=(h2a2+k2b2+l2c2)12

**Figure 2:**
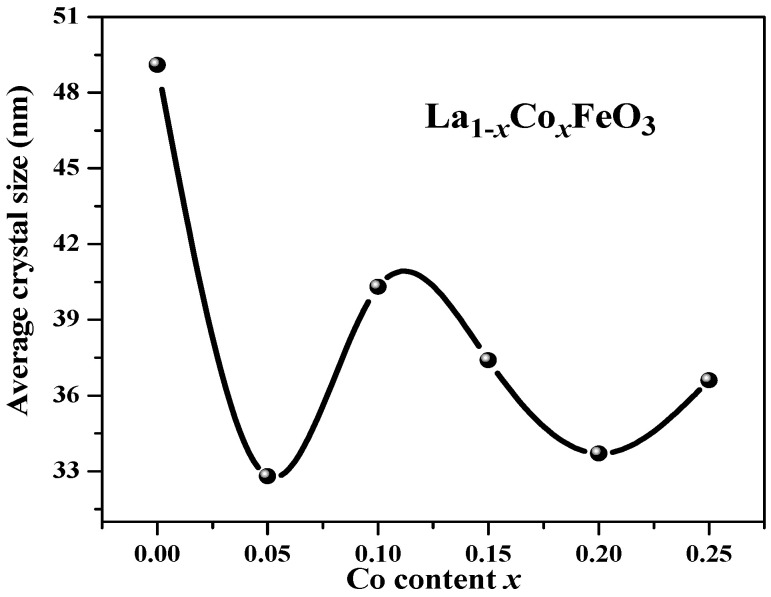
Average grain size of La_1*−x*_Co*_x_*FeO_3_ (*x* = 0~0.25) calcined at 700 °C for 6 h.

**Figure 3:**
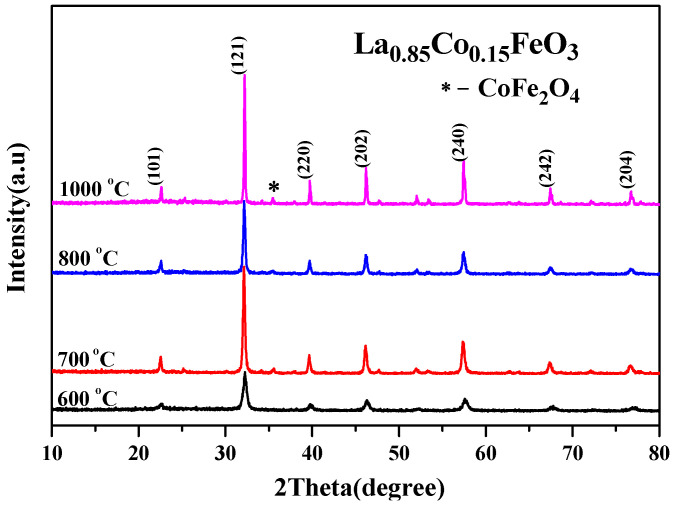
XRD diffraction pattern of La_0.85_Co_0.15_FeO_3_ calcined at 600~1000 °C for 2 h.

Figure 2 shows the change trend of the average grain size obtained via jade software. After doping Co^3+^, the diffraction peak was wide, and the grain size decreased. When the doping amount *x* > 0.05, the average grain size changed irregularly with the increase in x value. This may be related to the second phase CoFe_2_O_4_ [3]. Figure 3 is an XRD diffraction pattern of the La_0.85_Co_0.15_FeO_3_ sample. When the calcination temperature is 1000 °C, XRD detects impurities CoFe_2_O_4_. Rare earth transition metal composite oxide LaFeO_3_ material is a typical perovskite orthorhombic structure and a p-type semiconductor [5,6]. The rare earth ion La^3+^ with relatively large ion radius is more likely to occupy position A, which is located in the hole composed of FeO_6_ octahedron, whereas the transition metal ion Fe^3+^ with relatively small ion radius is more likely to occupy position B [7,12]. Fe^3+^ and the surrounding six O^2™^ form a FeO_6_ octahedron structure [17].

### 2.2. XRD and TG-DTA Analysis of La_1−x_R_x_FeO_3_ (R = Al)

**Figure 4:**
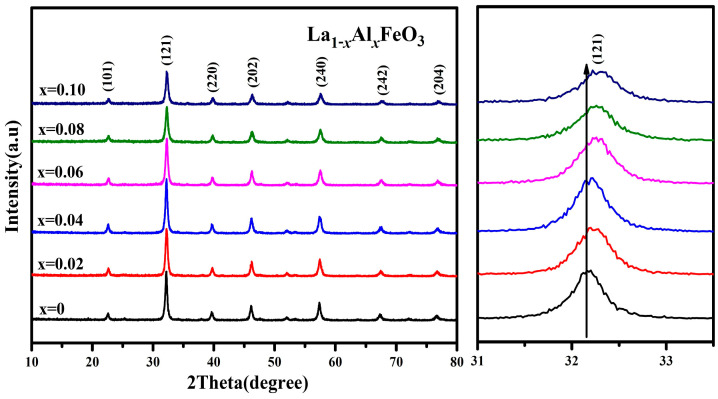
XRD diffraction pattern of La_1*−x*_Al*_x_*FeO_3_ (*x* = 0~0.10) calcined at 600 °C for 2 h.

Figure 4 is the XRD pattern of the La_1*−x*_Al*_x_*FeO_3_ (*x* = 0~0.10) sample. This may be due to the fact that the Al^3+^ ionic radius (0.0535 nm) is similar to the B-site Fe^3+^ ionic radius (0.078 nm), resulting in the formation of the Fe_2_O_3_ impurity phase. Table 1 shows that the lattice parameters (a, b, c), crystal cell volume and particle size showed a trend of being smaller with the increase in Al^3+^ doping concentration because La^3+^ ion radiuses (0.274 nm) are replaced by a smaller Al^3+^ ion radiuses [25].

Table 1 shows that as the Al^3+^ ion doping amount increased, the cell volume decreased. It was estimated using Scherrer’s formula:

(2) $$D=\frac{K\cdot \gamma }{\beta \cdot \cos\theta }$$

The XRD pattern clearly shows that the diffraction peak drifted in the direction of a larger angle, which can also indicate that the lattice parameters and the unit cell volume have a tendency to decrease.

**Figure 5:**
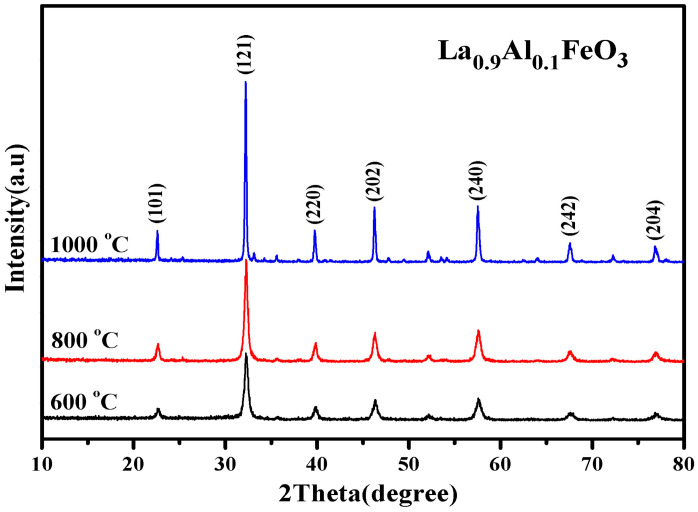
XRD diffraction pattern of La_0.9_Al_0.1_FeO_3_ samples calcined at temperatures between 600 and 1000 °C for 2 h.

Figure 5 is the XRD diffraction pattern of La_0.9_Al_0.1_FeO_3_ samples. When the calcination temperature was 800 °C, Fe_2_O_3_ peaks began to appear. Table 2 presents the lattice parameters of the La_0.9_Al_0.1_FeO_3_ sample calcined at 600 °C, 800 °C, and 1000 °C. This explains that according to the equation below, the smaller radius of Al^3+^ (ionic radius of 0.182 nm) replaced the larger radius of La^3+^ (ionic radius of 0.274 nm). At the same time, to maintain the charge balance, Fe^3+^ was oxidized to Fe^4+^ or oxygen vacancies appeared, resulting in lattice distortion.

**Figure 6:**
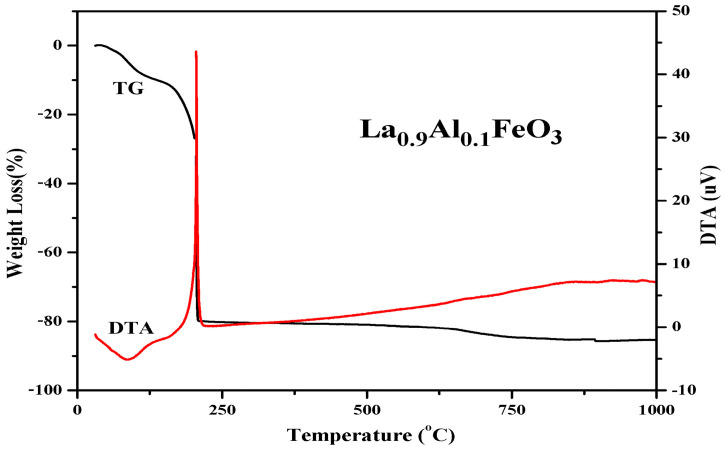
TG and DTA curves of La_0.9_Al_0.1_FeO_3_ xerogel.

Figure 6 is the TG and DTA curves of La_0.9_Al_0.1_FeO_3_ xerogel. When the temperature rose from 30 °C to 140 °C, the TG curve showed that the weight loss rate was about 10%. Then, at 90 °C, the DTA curve shows a weak endothermic peak due to water evaporation from the wet gel and the expulsion of water inside the dry gel (adsorbed water, crystallization water, and water vapor generated during the reaction). When the temperature increases from 140 °C to 207 °C, there is a weight loss of about 70 percent and a sharp exothermic peak corresponding to the DTA curves at about 207 °C. When the temperature is higher than 207 °C, the weight loss rate was less than 5%, forming La_1*−x*_Al*_x_*FeO_3_ [7].

### 2.3. XRD and TG-DTA Analysis of La_1−x_R_x_FeO_3_ (R = Nd)

**Figure 7:**
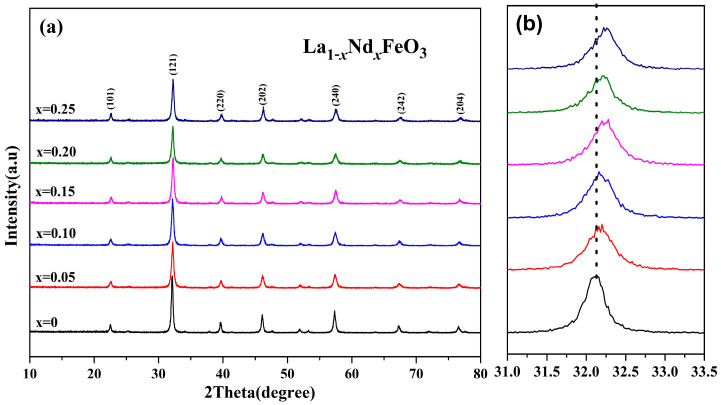
(**a**) XRD of La_1*−x*_Nd*_x_*FeO_3_ (*x* = 0~0.25) samples calcined at 600 °C and (**b**) (121) peak drift.

Figure 7 shows the XRD diffraction pattern of La_1*−x*_Nd*_x_*FeO_3_ (*x* = 0~0.25) samples calcined at 600 °C for 2 h and (121) peak drift pattern. According to Figure 7b, the (121) diffraction peak slightly increased with the increase in Nd^3+^ doped concentration and drifted in the direction of 2θ [24]. The substitution of small radius Nd^3+^ (ionic radius of 0.127 nm) for larger radius La^3+^ (ionic radius of 0.274 nm) leads to lattice distortion [26]. The lattice parameters of corresponding samples are as shown in Table 3. As the Nd^3+^ content increased, the crystal lattice parameters (a, b, and c) changed accordingly [27], and the cell volume of the samples decreased. The reason is that the La ion was gradually replaced by Nd ion, which reduced the average ionic radius of A-bit and, in turn, caused cell shrinkage, resulting in a decrease in cell volume [28]. The grain size of the samples can be estimated according to Scherrer’s formula. When *x* ≤ 0.05, the average grain size decreased. When *x* > 0.05, as the doping amount increased, the grain size increased. It is possible that the ion size of A-bit changed with its nearest neighbor, and the size of the neighboring oxygen atoms depends on the grain size [29,30].

**Figure 8:**
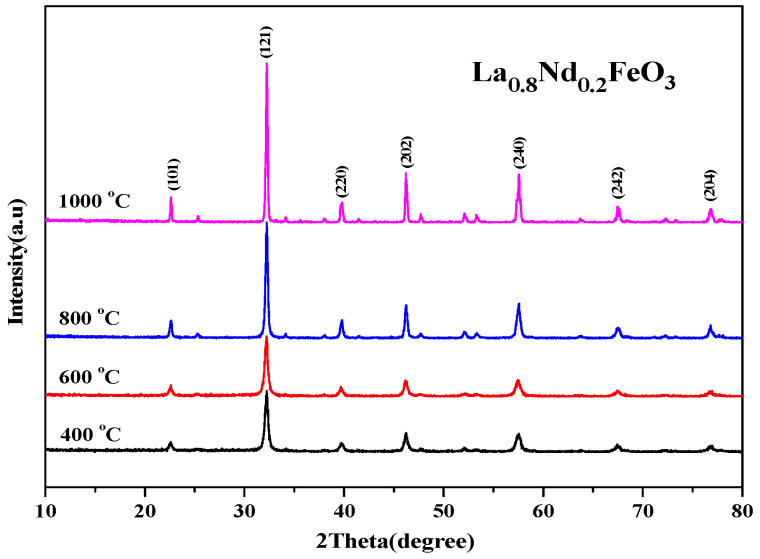
XRD diffraction pattern of La_0.8_Nd_0.2_FeO_3_ samples calcined at between 400 and 1000 °C for 2 h.

The XRD diffraction pattern in Figure 8 shows that the main diffraction peak is consistent with the standard sample LaFeO_3_ card (JCPDS No. 37-1493). No other phases were generated, and the space group is Pnma.

The results show that under four calcination conditions, Nd^3+^ is better immersed in the crystal lattice of the perovskite, and all characteristic peaks have been indexed according to the orthorhombic structure [1]. As the calcination temperature rose, the diffraction peak became sharper, the half-height width decreased, and the average grain size estimated by Scherrer’s formula gradually increased. The results show that the calcination temperature had a direct effect on the grain size of the powder when the samples were synthesized using the sol-gel method. The higher the calcination temperature, the higher the energy, the larger the grain size, and the larger the size of the powder.

### 2.4. XRD and TG-DTA Analysis of La_1−x_R_x_FeO_3_ (R = Sm)

**Figure 9:**
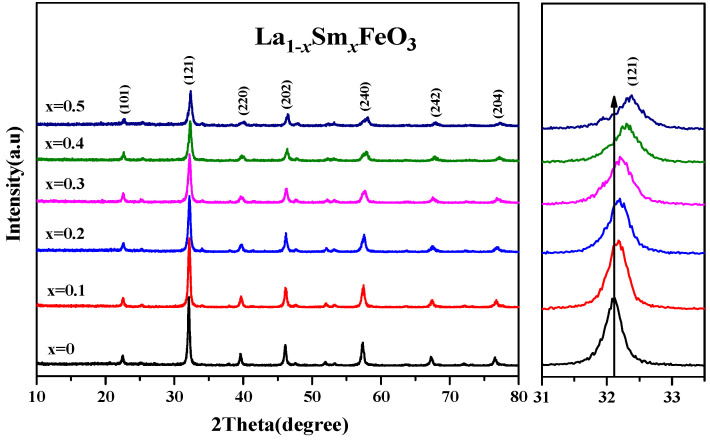
XRD of La_1*−x*_Sm*_x_*FeO_3_ (*x* = 0~0.5) and the peak drift (121).

Figure 9 shows an XRD diffraction pattern of the La_1*−x*_Sm*_x_*FeO_3_ (*x* = 0~0.5) samples. According to the tolerance factor t = r_A_ + r_O_/1.414 (r_B_ + r_O_), r_A_, r_B_, and r_O_ are A-bit ionic radius, B-bit ionic radius, and O ionic radius, respectively. For part of the A-bit doped samples, r_A_ = (A′ ionic radius) (1 − *x*) + (radius of doped ions) *x*; when t values were between 0.75 and 1.00, the perovskite structure was stable. According to the ionic radius records [31,32], La^3+^ ionic radius was 0.274 nm, Sm^3+^ ionic radius was 0.124 nm, Fe^3+^ ionic radius was 0.0645 nm, O ionic radius was 0.132 nm, and the t value of the series samples was between 0.75 and 1.00, which had a stable perovskite structure [33,34]. As the Sm^3+^ doping concentration increased, the intensity of diffraction peaks decreased. The drift pattern shows that as the Sm^3+^ doping content increased, the diffraction peak drifted in the direction of the larger angle. This explains that according to the equation below, the smaller radius of Sm^3+^ (ionic radius of 0.124 nm) replaced the larger radius of La^3+^ (ionic radius of 0.274 nm).

**Figure 10:**
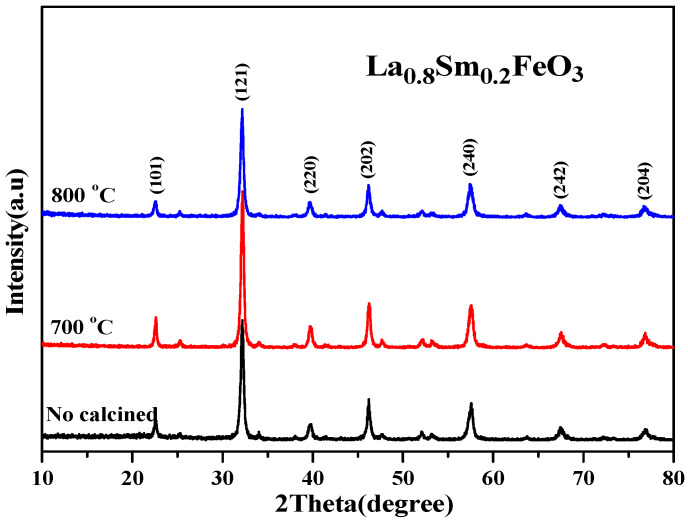
XRD diffraction pattern of uncalcined La_0.8_Sm_0.2_FeO_3_ samples that were then calcined at 700 °C and 800 °C for 2 h.

Table 4 shows that as the Sm^3+^ ion doping amount increased, the cell volume decreased. Figure 10 shows the XRD diffraction pattern of uncalcined La_0.8_Sm_0.2_FeO_3_ samples, and then calcined at 700 °C and 800 °C for 2 h. The sample diffraction peaks were sharp, suggesting that the samples crystallized well after sintering at a certain temperature. The half-width of the diffraction peak of the samples was 0.371, 0.333, and 0.378, respectively, corresponding to the aforementioned calcination temperatures, with a first decreasing then increasing change trend [1,2]. The average grain size of the samples was 22.7, 25.9, and 21.1 nm, respectively, corresponding to the aforementioned calcination temperatures, with a first increasing then decreasing change trend [6,7].

**Figure 11:**
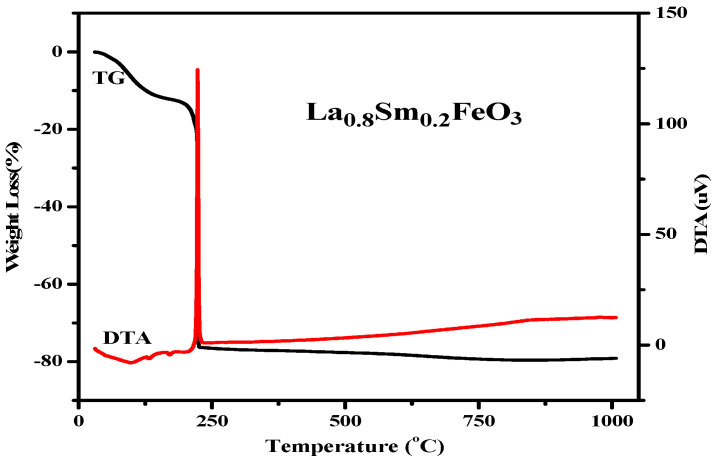
TG and DTA curves of La_0.8_Sm_0.2_FeO_3_ xerogel.

Figure 11 shows the TG and DTA curves of La_0.8_Sm_0.2_FeO_3_ xerogel. When the temperature rose from 30 °C to 200 °C, the TG curve showed that the weight loss rate was about 13%. Then, the DTA curve showed a weak endothermic peak at 98.5 °C due to the water evaporation from the wet gel. The weak endothermic peaks at 133 °C and 171 °C were due to the expulsion of water from inside the dry gel. When the temperature rose from 200 °C to 226 °C, the weight loss was about 63%, showing a sharp exothermic peak corresponding to the DTA curves at about 226 °C, which may be due to emissions of nitrates and organic substances, including NO*_x_*, CO*_x_*, and H_2_O. When the temperature was higher than 226 °C, the weight loss rate was less than 3%, indicating the formation of La_1*−x*_Sm*_x_*FeO_3_.

### 2.5. Magnetic Analysis of La_1−x_R_x_FeO_3_ (R = Co)

**Figure 12:**
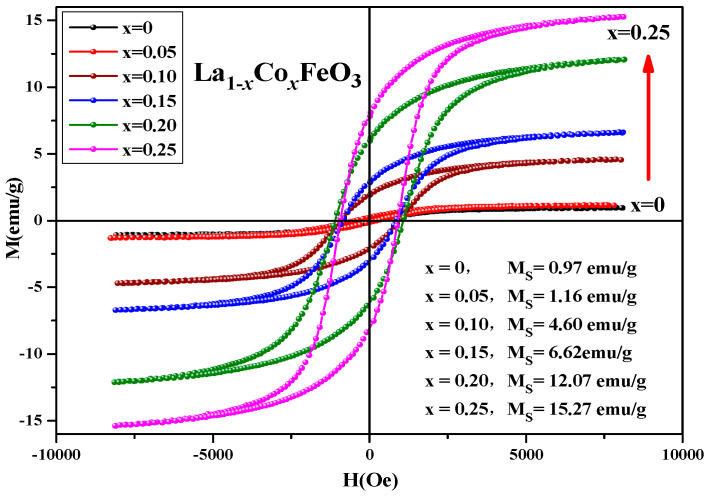
Hysteresis loop of La_1*−x*_Co*_x_*FeO_3_ (*x* = 0~0.25) samples calcined at 700 °C for 6 h.

Figure 12 shows the hysteresis loop of the La_1*−x*_Co*_x_*FeO_3_ (*x* = 0~0.25) sample. When the applied magnetic field is 8000 Oe, the magnetization reaches a saturation state [35]. As shown in Table 5, with the increase in Co^2+^ concentration, the magnetic loop area and magnetization increased. The change of valence state of the A-site ions will directly affect the state of the oxygen ions, which will eventually lead to the generation of oxygen vacancies.

**Figure 13:**
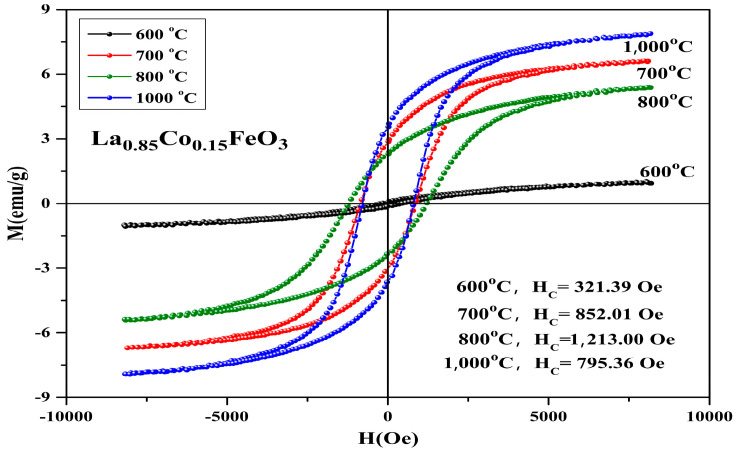
Hysteresis loop of La_0.85_Co_0.15_FeO_3_ samples calcined at 600~1000 °C for 6 h.

Figure 13 shows the hysteresis loop of La_0.85_Co_0.15_FeO_3_ sample calcined at temperatures between 600 and 1000 °C for 6 h. When the applied magnetic field is 8000 Oe, the magnetization reaches a saturation state. Figure 13 and Table 6 show that when the samples calcined temperature rose from 600 °C to 700 °C, the loop area of the M-H curve changed significantly. With the increase in calcination temperature, the saturation magnetization, remanent magnetization, and coercivity of the samples increased, which may be affected by the mixed-phase CoFe_2_O_4_ samples.

**Figure 14:**
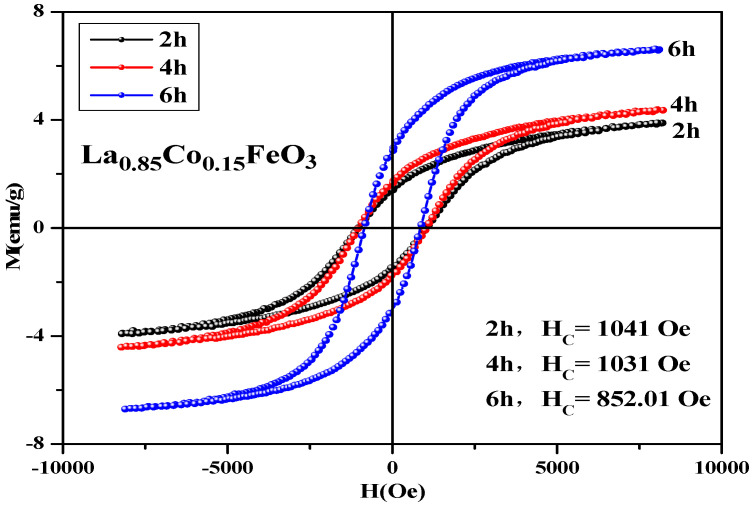
Hysteresis loop of La_0.85_Co_0.15_FeO_3_ samples calcined at 700 °C for 2 h, 4 h, and 6 h.

In Table 7, Co^2+^ doping improves the coercive force of a sample. It is caused by the relation of coercive force Hc and magnetocrystalline anisotropy [18,19]. The magnetic moments of the sublattice array composed of iron ions are oppositely aligned in the same straight line, so the samples show antiferromagnetic properties on a macroscopic scale. The magnetic ion Co has a 3d^7^ electronic configuration and stronger spin-orbit coupling. When Co^2+^ replaced A-bit non-magnetic ion La^3+^, the La_1*−x*_Co*_x_*FeO_3_ samples had a stronger magnetocrystalline anisotropy constant. When the doping amount *x* ≥ 0.10, the changes in the coercivity were irregular. There may be a relationship between coercivity and grain size [36]. When the calcination temperature was 600 °C, no CoFe_2_O_4_ mixed phase was detected. When it rose to 700 °C, we found the second phase CoFe_2_O_4_, and when it rose to 1000 °C, the magnetization of the sample was the largest and the coercive force of sample was 1213 Oe. Figure 14 shows the hysteresis loop of La_0.85_CO_0.15_FeO_3_ samples. The calcination time can regulate the magnetic properties of the sample, as shown in Table 7. From 2 h to 6 h, the magnetic properties reduced significantly.

### 2.6. Magnetic Analysis of La_1−x_R_x_FeO_3_ (R = Al)

**Figure 15:**
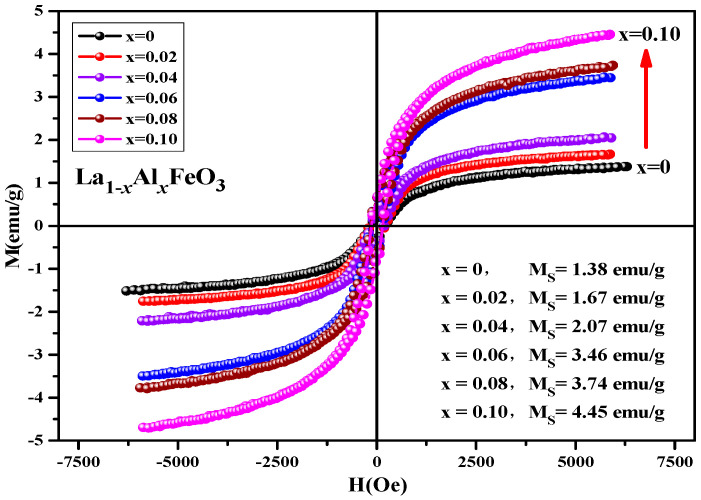
Hysteresis loop of La_1*−x*_Al*_x_*FeO_3_ samples calcined at 600 °C for 2 h.

**Figure 16:**
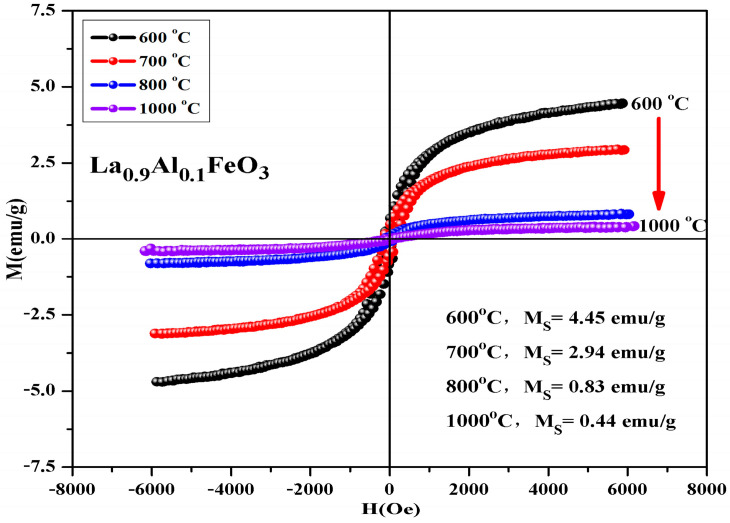
Hysteresis loop of La_0.9_Al_0.1_FeO_3_ samples calcined at between 400 and 1000 °C for 2 h.

Figure 15 is hysteresis loop of La_1*−x*_Al*_x_*FeO_3_ (*x* = 0~0.10) samples, the magnetic loop area and magnetization increased with the increase in Al^3+^ ion concentration and the weak ferromagnetic properties are shown in Table 8. In fact, in the perovskite structure, Fe^3+^ was in a distorted octahedral B-site location, and the octahedron distortion was inclined along the c-axis direction, and the inclination depended on the size of the adjacent A-site ions, which ultimately determined the Fe-O-Fe super-exchange angle. The reason this may enhance the magnetic properties is as follows: First, when Al^3+^ ions substituted La^3+^ ions, the effective size of the A-site of octahedral reduced, changing the Fe-O-Fe super-exchange angle and promoting super-exchange interaction. Second, when the sample was doped, only a small fraction of La^3+^ was substituted by Al^3+^ ions, which tend to occupy Fe^3+^ ion positions at octahedral B-bit. When Al^3+^ ion substituted the Fe^3+^ ion, Fe^3+^ was squeezed into the La^3+^ ion position of the regular tetrahedron A-bit. This time, the Fe^3+^ ion spin was not compensated, and the magnetic properties of the sample can be improved. In addition, Fe_2_O_3_ impurity phase was detected by XRD, which may also enhance the magnetic properties of the La_1*−x*_Al*_x_*FeO_3_ samples [21,22]. Figure 16 shows the hysteresis loop of La_0.9_Al_0.1_FeO_3_ samples. It can be seen from the figure that as the calcination temperature increased, the M-H curve of the sample enclosed area reduced, the ferromagnetic order gradually weakened and tended to be of the antiferromagnetic order, which may be due to with the increase in sintering temperature, decrease in iron oxide composition, and changes in the magnetic properties [14].

**Figure 17:**
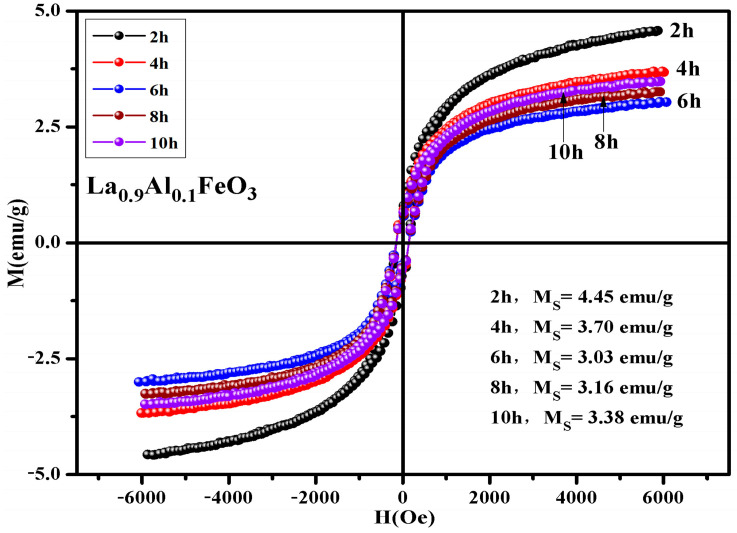
Hysteresis loop of La_0.9_Al_0.1_FeO_3_ samples calcined at 600 °C between 2 and 10 h.

Table 9 shows that the calcination temperature has the same effect on Ms, Mr, and Hc; all of which showed a declining trend as T increased. Figure 17 shows the hysteresis loop of La_0.9_Al_0.1_FeO_3_ samples. The calcination time can regulate the magnetic properties of the sample, as shown in Table 10. From 2 h to 6 h, the magnetic properties reduced significantly. When the calcination time increased from 6 h to 10 h, the sintering time showed little impact on the changes in the magnetic properties.

### 2.7. Magnetic Analysis of La_1−x_R_x_FeO_3_ (R = Nd)

**Figure 18:**
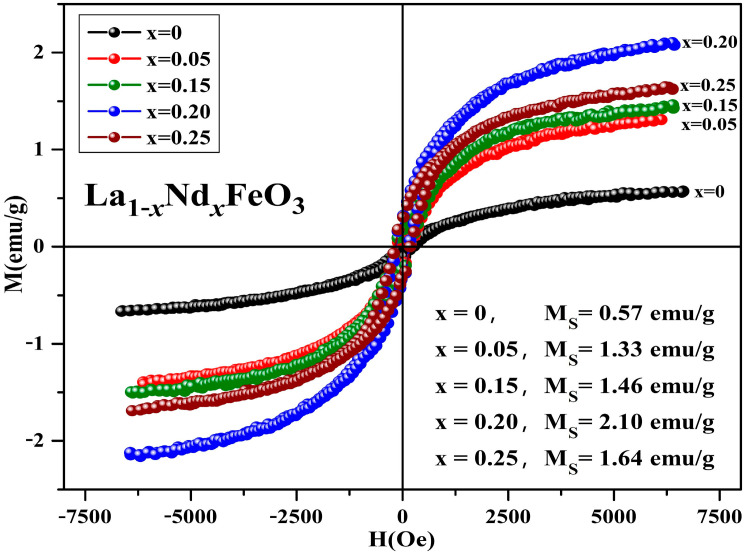
Hysteresis loop of La_1*−x*_Nd*_x_*FeO_3_ (*x* = 0~0.25) samples calcined at 600 °C for 2 h.

Figure 18 is the hysteresis loop of the La_1*−x*_Nd*_x_*FeO_3_ (*x* = 0~0.25) samples calcined at 600 °C for 2 h, which was measured at room temperature, and has an applied magnetic field of 0.6 T. It can be seen that the hysteresis loop had a narrow shape, and all samples did not reach a saturation state, showing weak ferromagnetic properties. When x ≤ 0.20, as the Nd^3+^ doped concentration increased, the magnetization of sample increased. This may be due to the fact that the Nd atomic magnetic moment is not zero. The introduction of Nd^3+^ leads to an unbalanced distribution of magnetic moment of the whole structure, thereby increasing the magnetization of the samples. When Nd^3+^ was doped, the magnetization of sample began to decrease.

The Nd^3+^ ion concentration reached a certain amount, the Fe-O bond length decreased, and the inclination of the octahedron decreased, thereby strengthening antiferromagnetic exchange interaction and weakening the interaction between weak ferromagnetics. Eventually, the magnetization of the samples was weakened. Table 11 shows the magnetic parameters of the samples. As the Nd^3+^ content increased, the remanent magnetization and saturation magnetization of the samples first increased then decreased. The magnetic moment and ionic radius of rare earth elements ions are the main factors affecting the saturation magnetization of the doping samples [37]. The coercivity of the samples shows an upward trend, and the main factors affecting the coercivity of the samples include magnetic anisotropy, grain size, micro strain, stress, crystal symmetry and spin-orbit coupling effect, magnetic single domain size, and impurities [31].

**Figure 19:**
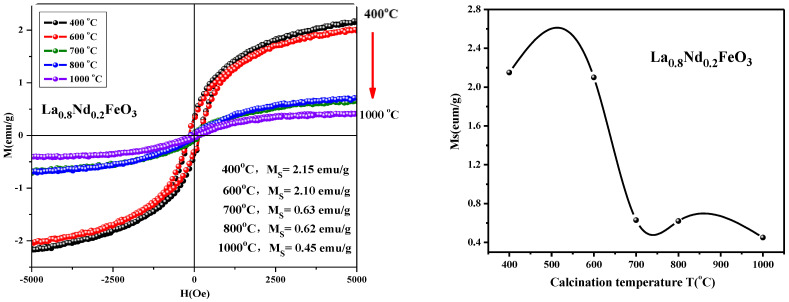
Hysteresis loop and saturation magnetization of La_0.8_Nd_0.2_FeO_3_ samples calcined between 400 and 1000 °C for 2 h.

**Figure 20:**
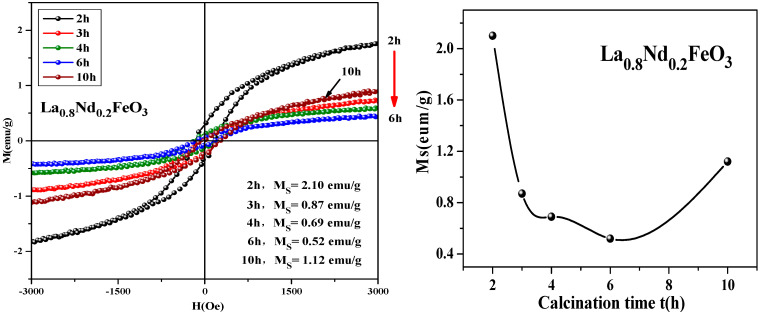
Hysteresis loop and saturation magnetization of La_0.8_Nd_0.2_FeO_3_ samples calcined at 600 °C for different durations.

From Figure 19, it can be seen that the magnetization of the samples hardly changed when the calcination temperature rose from 400 °C to 600 °C. As the calcination temperature decreased, the saturation magnetization (Ms) of the samples decreased. The magnetization of the samples changed little when the temperature continued to rise to 1000 °C. As the calcination temperature increased, the magnetization of the samples decreased. It can be seen from Figure 20 that as the calcination time extended, the saturation magnetization of the samples first decreased then increased. When the calcination time was extended, the magnetization changed significantly, and the optimum calcination time was 2 h. The main factors affecting the coercivity of the samples include magnetic anisotropy, grain size, micro strain, stress, crystal symmetry and spin-orbit coupling effect, magnetic single domain size, impurities, and calcination temperature.

### 2.8. Magnetic Analysis of La_1−x_R_x_FeO_3_ (R = Sm)

**Figure 21:**
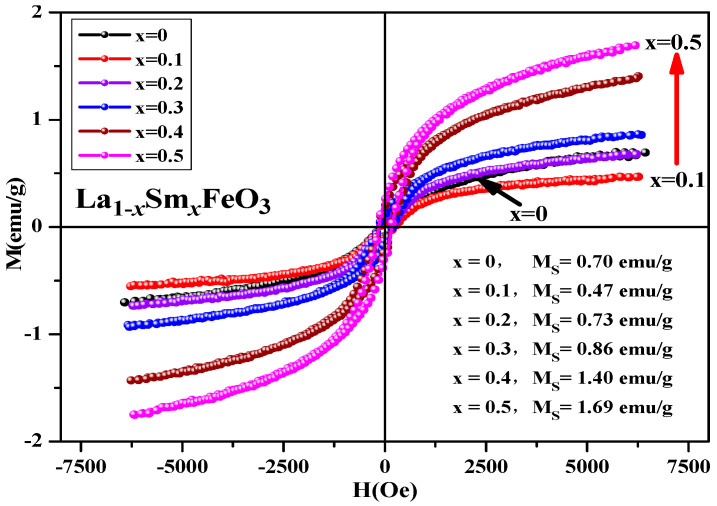
Hysteresis loop of uncalcined La_1*−x*_Sm*_x_*FeO_3_ (*x* = 0~0.5) samples.

Figure 21 shows the hysteresis loop of the uncalcined La_1*−x*_Sm*_x_*FeO_3_ (*x* = 0~0.5) samples. It can be seen that the hysteresis loop had a narrow shape, and all samples did not reach a saturation state, showing weak ferromagnetic properties. Table 12 shows the distribution of magnetic parameters of the sample. This may be because the Sm^3+^ ions are magnetic in nature [37], and together with Fe^3+^ and O^2−^, generate a super-exchange interaction, which affected the net magnetic moment size of the crystals, ultimately affecting the magnetization of the samples.

## 3. Experimental

Compared with other preparation methods, the advantages of the sol-gel method are as follows [13,14]: (1) The uniformity of components in the reaction process is good, and the ratio of reactive ions is controllable [15,16]; (2) It is easy to obtain smaller particle size, better dispersion, and higher purity [17,18]; (3) The experimental operation is simple and the reaction temperature is low [19,20]. La_1*−x*_R*_x_*FeO_3_ (R = Co, Al, Nd, Sm) was synthesised by the sol-gel combustion method. The synthetic raw material of the sample is analytically pure nitrate La(NO_3_)_3_·nH_2_O, Fe(NO_3_)_3_·9H_2_O, Co(NO_3_)_3_·6H_2_O, Al(NO_3_)_3_·9H_2_O, Sm(NO_3_)_3_·6H_2_O, Nd(NO_3_)_3_·6H_2_O ammonia (NH_3_·H_2_O), and citric acid (C_6_H_8_O_7_·H_2_O). The crystalline structure was investigated using X-ray diffraction (D/max-2500V/PC, Rigaku). Magnetization measurements were carried out with vibrating sample magnetometer (VSM-100) at room temperature.

## 4. Conclusions

The effects of sol gel self propagating synthesis method, treatment temperature and doping amount of rare earth ions were studied in this paper. The doping relative sample shape, particle size, microstructure, and magnetic properties were also discussed. In this paper, the synthesis of LaFeO_3_ nanoparticles doped with Co^2+^, Al^3+^, Nd^3+^, and Sm^3+^ ion doping LaFeO_3_ on the structure and magnetic properties of La_1*−x*_R*_x_*FeO_3_ (R = Co, Al, Nd, Sm). The diffraction peak intensity is decreased with the increase in Co^2+^ doping concentration. The crystallinity of the sample is reduced. Fe^3+^ was oxidized to Fe^4+^, or oxygen vacancies appeared, resulting in lattice distortion. When Co^2+^ replaced A-bit non-magnetic ion La^3+^, the La_1*−x*_Co*_x_*FeO_3_ sample will obtain a larger magnetocrystalline anisotropy constant. TG and DTA analysis show that when the temperature is higher than 207 °C, La_1*−x*_R*_x_*FeO_3_ (R = Al) is formed. Magnetic properties show that La_1*−x*_Al*_x_*FeO_3_ has a weak ferromagnetism; with increasing Al^3+^ concentration, magnetic properties of the samples showed an increasing trend, and the sintering time that continues to extend with the change of the magnetic properties has a little influence. The results of XRD analysis show that Nd^3+^ substitutes the perovskite La to form a solid La_1*−x*_Sm*_x_*FeO_3_ solution, which is a single orthogonal perovskite structure. Through calcination at different temperatures, La_0.8_Nd_0.2_FeO_3_ has a pure phase. The peak of Fe-O-Fe bond antisymmetric stretching vibration in the FeO_6_ regular octahedron was at about 569 cm^−1^. The hysteresis loop of La_0.8_Nd_0.2_FeO_3_ samples calcined at temperatures between 400 and 1000 °C for 2 h, respectively, show that as the temperature increased, the saturation magnetization decreased, and the maximum value and minimum value of the saturation magnetization was 2.15 emu/g and 0.45 emu/g, respectively. As the calcination time increased, the saturation magnetization first decreased then increased, and the minimum value of the saturation magnetization was 0.52 emu/g. The results of XRD analysis show that Nd^3+^ substitutes the perovskite La entering the lattice to form La_1*−x*_Sm*_x_*FeO_3_ solid solution, which is a single orthogonal perovskite structure with a space group of Pnma, and no impurity peaks were observed. In the future, the reaction mechanism of the sol-gel process can be studied to improve the magnetic properties of LaFeO_3_ nanoparticles.

## Figures and Tables

**Table 1:** Lattice parameters of La_1*−x*_Al*_x_*FeO_3_ (*x* = 0~0.10) samples calcined at 600 °C for 2 h.

Content (*x*)	a (Å)	b (Å)	c (Å)	Vol (Å^3^)	Density	Crystallite (nm)
0	5.56631	7.86474	5.55496	243.18	6.6303	22
0.02	5.55067	7.85841	5.55787	242.43	6.6509	21
0.04	5.55676	7.85729	5.55981	242.75	6.6422	20
0.06	5.54878	7.84785	5.55199	241.77	6.6692	21
0.08	5.54461	7.84311	5.55592	241.61	6.6735	18
0.1	5.5407	7.84001	5.54634	240.93	6.6924	18

**Table 2:** Lattice parameters of La_0.9_Al_0.1_FeO_3_ sample calcined at 600 °C, 800 °C, and 1000 °C for 2 h.

Temperature (°C)	a (Å)	b (Å)	c (Å)	Vol (Å^3^)	Density	Crystallite (nm)
600	5.5407	7.84001	5.54634	240.93	6.6924	18
800	5.54272	7.84423	5.55089	241.34	6.6808	21
1000	5.55049	7.85035	5.54689	241.7	6.6711	66

**Table 3:** Lattice parameters of La_1*−x*_Nd*_x_*FeO_3_ (*x* = 0~0.25) calcined at 600 °C for 2 h.

Content (*x*)	a (Å)	b (Å)	c (Å)	Vol (Å^3^)	Density	Crystallite (nm)
0	5.56716	7.87553	5.56760	244.11	6.6052	30
0.05	5.56559	7.86565	5.55402	243.14	6.6315	21
0.10	5.56556	7.86231	5.54953	242.84	6.6397	21
0.15	5.55551	7.85002	5.55155	242.11	6.6598	23
0.20	5.55488	7.85779	5.56101	242.73	6.6426	23
0.25	5.54714	7.84794	5.55431	241.80	6.6683	23

**Table 4:** Lattice parameters of uncalcined La_1*−x*_Sm*_x_*FeO_3_ (*x* = 0~0.5).

x	a (Å)	b (Å)	c (Å)	Vol (Å^3^)	Density (g/cm^3^)	Crystallite (nm)
0	5.56723	7.87386	5.56872	244.11	6.6052	29
0.1	5.56808	7.85908	5.55037	242.88	6.6385	27
0.2	5.53643	7.85412	5.58219	242.73	6.6426	24
0.3	5.53733	7.85037	5.56868	242.07	6.6608	20
0.4	5.57219	7.83427	5.51067	240.56	6.7025	19
0.5	5.60195	7.81511	5.4613	239.09	6.7437	19

**Table 5:** Magnetic parameters of La_1*−x*_Co*_x_*FeO_3_ sample calcined at 700 °C.

Content (*x*)	0	0.05	0.10	0.15	0.20	0.25
Ms (emu/g)	0.97	1.16	4.60	6.62	12.07	15.27
Mr (emu/g)	0.05	0.19	1.91	2.83	5.91	7.70
Hc (Oe)	92.51	347.27	944.62	852.01	1087	921.26

**Table 6:** Magnetic parameters of La_0.85_Co_0.15_FeO_3_ sample calcined between 600 and 1000 °C.

Temperature (°C)	600	700	800	1000
Ms (emu/g)	1.01	6.62	5.39	7.88
Mr (emu/g)	0.12	2.83	2.28	3.51
Hc (Oe)	321.39	852.01	1213	795.36

**Table 7:** Magnetic parameters of La_0.85_CO_0.15_FeO_3_ sample calcined at 700 °C.

Time (Hour)	2 h	4 h	6 h
Ms (emu/g)	3.89	4.38	6.62
Mr (emu/g)	1.39	1.65	2.83
Hc (Oe)	1041	1031	852.01

**Table 8:** Magnetic parameters of La_1*−x*_Al*_x_*FeO_3_ (*x* = 0~0.10) samples calcined at 600 °C for 2 h.

Content (*x*)	0	0.02	0.04	0.06	0.08	0.10
Ms (emu/g)	1.38	1.67	2.07	3.46	3.74	4.45
Mr (emu/g)	0.20	0.34	0.32	0.58	0.60	0.63
Hc (Oe)	178.05	196.65	167.01	156.40	151.11	140.21

**Table 9:** Magnetic parameters of La_0.9_Al_0.1_FeO_3_ samples calcined at temperatures between 600 and 1000 °C for 2 h.

Temperature (°C)	600	700	800	1000
M_s_ (emu/g)	4.45	2.94	0.83	0.44
Mr (emu/g)	0.63	0.45	0.09	0.02
Hc (Oe)	140.21	146.65	131.46	115.69

**Table 10:** Magnetic parameters of La_0.9_Al_0.1_FeO_3_ samples calcined at 600 °C for between 2 and 10 h.

Time (Hour)	2 h	4 h	6 h	8 h	10 h
M_s_ (emu/g)	4.45	3.70	3.03	3.16	3.38
Mr (emu/g)	0.63	0.63	0.50	0.49	0.53
Hc (Oe)	140.21	177.76	147.03	145.80	145.55

**Table 11:** Magnetic parameters of La_1*−x*_Nd*_x_*FeO_3_ (*x* = 0~0.25) samples calcined at 600 °C for 2 h.

Content (*x*)	0	0.05	0.15	0.20	0.25
Ms (emu/g)	0.57	1.33	1.46	2.10	1.64
Mr (emu/g)	0.04	0.13	0.17	0.30	0.28
Hc (Oe)	148.15	153.51	168.90	159.21	179.53

**Table 12:** Magnetic parameters of uncalcined La_1*−x*_Sm*_x_*FeO_3_ (*x* = 0~0.5) samples.

Content (*x*)	0	0.1	0.2	0.3	0.4	0.5
Ms(emu/g)	0.7	0.47	0.73	0.86	1.4	1.69
Mr (emu/g)	0.05	0.06	0.08	0.13	0.19	0.25
H_C_ (Oe)	145.95	181.19	168.12	172.41	163.57	158.89

## Data Availability

Not applicable.

## Sample Availability

Samples of the compounds are available from the authors.
